# Laparoscopic varicocelectomy in male infertility: Improvement of seminal parameters and effects on spermatogenesis

**DOI:** 10.1007/s00508-021-01897-w

**Published:** 2021-06-28

**Authors:** Felix Seiler, Philipp Kneissl, Claudius Hamann, Klaus-Peter Jünemann, Daniar Osmonov

**Affiliations:** Department of Urology, University Medical Center Schleswig-Holstein, Campus Kiel, Arnold-Heller-Str. 3, 24105 Kiel, Germany

**Keywords:** Varicocele, Laparoscopy, Sterility, Azoospermia, Assisted reproductive techniques

## Abstract

**Background:**

The suitability of laparoscopic varicocelectomy for assisted reproductive technology depends on the improvement of semen parameters. The present study analyzed the improvement of semen parameters following laparoscopic varicocele ligation.

**Material and methods:**

A retrospective study of the laparoscopic varicocele clippings at the Department of Urology of University Hospital of Kiel between the years 2007 and 2019 was conducted. The semen analyses according to WHO standards (sperm count, density, motility and morphology) were conducted before and 12 months after surgery. Screening for surgical complications took place at the time of the follow-up seminal analysis. Included were patients with oligozoospermia, asthenozoospermia and/or teratozoospermia (group 1, OAT) or with nonobstructive azoospermia (group 2, NOA).

**Results:**

This study included data of 27 patients and 22 patients presented preoperative OAT (81%, group 1). Another 5 patients showed NOA (19%, group 2). Data of group 1 showed that semen parameters normalized in 32% of the patients after surgery. Significant improvement in total sperm count (*p* < 0.005), sperm density (*p* < 0.005) and total motile sperm count (*p* < 0.005) was observed. No deterioration of semen parameters was observed. In group 2 we detected spermatozoa in 1 case in the postoperative ejaculate. None of the patients showed complications according to the Clavien-Dindo classification, postoperative hydrocele formation or recurrence of varicocele at the time of control spermiogram.

**Conclusion:**

Laparoscopic varicocelectomy is a valid therapeutic approach to improve semen parameters for further assisted reproductive techniques. Spermatogenesis may be induced for patients with NOA. Normalization of semen parameters can be achieved for patients with OAT.

## Introduction

A varicocele is the dilation of the pampiniform plexus. The incidence of varicocele in the general population is between 4.4% and 22.6% but as many as 21–41% of men evaluated for primary infertility have varicoceles and of patients with secondary infertility the incidence of varicoceles is 75–81% [1]. The apparent predisposition for the development of varicoceles on the left side has been explained by the anatomy of the left internal spermatic vein, draining perpendicularly into the left renal vein and by valvular abnormalities in the left internal spermatic vein [2]. The WHO classification of varicocele is based on the Dubin and Amelar classification (see Table 1; [3]).

**Table 1:** WHO classification of varicoceles [11]

	Physical examination
Subclinical	Not palpable, visible only in sonography
Grade 1	Palpable only during Valsalva maneuver
Grade 2	Palpable at rest but not visible
Grade 3	Visible and palpable at rest

An impairment of the testicular function by the varicocele is presumed. Etiologically, a reflux of toxic metabolites, an increase in scrotal temperature and a testicular tissue hypoxia are considered [4]. The deleterious effects of the varicocele on spermatogenesis seem to be progressive. The significantly increased incidence of varicoceles in patients with secondary infertility compared to the total population supports the hypothesis of progressive damage to spermatogenesis by varicocele. Nevertheless, a protective varicocele treatment in adolescents without pathological spermiogram findings does not seem to offer significant advantages for the patients. Thus, the optimal time of treatment remains the subject of current debate [5].

Varicoceles represent a challenge for clinical practice, as their role in the etiology of fertility disorders and the various treatment options have been the subject of controversial discussion for decades. According to European Association of Urology (EAU) guidelines, surgical treatment is indicated for adults with spermiogram alterations and male factor infertility [6]. The spermiogram differentiates between oligozoospermia, asthenozoospermia and teratozoospermia (see Table 2). If all three parameters are altered, the diagnosis is oligoasthenoteratozoospermia (OAT) syndrome. In the absence of sperm in the ejaculate, a distinction is made between obstructive and nonobstructive azoospermia. Regarding varicocele treatment, nonobstructive azoospermia (NOA) is of importance. For the treatment of male factor infertility there are various options of assisted reproductive technology. The aim of varicocele therapy in male infertility is to improve seminal parameters for assisted reproduction technologies (ART) and thus reduce the invasiveness of these measures [7].

**Table 2:** Lower reference limits for semen characteristics [11]

Parameter	Lower reference limit
Semen volume (ml)	≥ 1.5
Total sperm count (TSC) (10^6^ per ejaculate)	≥ 39
Sperm density (10^6^/ml)	≥ 15
Total motility (%)	≥ 40
Total motile sperm count (TMSC) (10^6^ per ejaculate)	≥ 12.5
Sperm morphology (normal forms) (%)	≥ 4

Several surgical approaches are available to accomplish a varicocelectomy. The originally open surgical treatment has been supplemented by microsurgical and laparoscopic techniques and by percutaneous varicocele embolization [8]. The current literature does not provide clear evidence of the superiority of any of the surgical procedures, although there is some evidence of the advantages of a microsurgical approach [9]. Each surgical technique has its advantages and disadvantages, and finally it is up to the surgeon to choose the technique that can be safely mastered and with few complications. In the Department of Urology at the University Medical Center Kiel, the technique of laparoscopic varicocelectomy was established in 2007 and has been used since then.

The present study analyzed the improvement of semen parameters following laparoscopic varicocele ligation. The alteration in seminal parameters after the procedure was examined for patients with reduced seminal parameters. Patients with NOA were examined postoperatively for sperm in the ejaculate and thus whether an induction of spermatogenesis occurred after the procedure.

## Patients, material and methods

For this purpose, we conducted a retrospective analysis of laparoscopic varicocele ligations at the University Medical Center Schleswig-Holstein, Campus Kiel, between 2007 and 2019. Patients with varicocele and spermiogram alterations were included in the analysis. Our study corresponds to a pre-test post-test design. According to their preoperative spermiogram findings, the patients were divided into two groups. Group 1 included patients with preoperative oligozoospermia and/or asthenozoospermia and/or teratozoospermia. Group 2 included patients with preoperative nonobstructive azoospermia.

The spermiogram results according to WHO criteria were examined before and 12 months after the surgery. Screening for surgical complications took place at the time of the follow-up seminal analysis. The preoperative assessment was performed by an experienced physician with the additional qualification in andrology according to a standardized protocol. The diagnosis included a physical examination to classify the varicocele according to WHO criteria and a sonographic examination [10].

For the present study, all patients examined gave the consent to the analysis of the data. In addition, the Ethics Committee of the Medical Faculty of the Christian-Albrechts-University of Kiel gave a positive vote (number D 568/18).

The laparoscopic varicocelectomy was performed with three ports in a conventional triangle formation. After installing the instruments, a retroperitoneal incision was made superior to the internal inguinal ring to separate the dilated testicular vein from testicular artery. Under preservation of the testicular artery the vein was ligated with two laparoscopic clips and cut.

The spermiograms were analyzed according to the WHO‑4 standard until 2010 and then according to the WHO‑5 standard [11, 12]. The changes in sperm concentration, total sperm count (TSC), sperm motility (progressive and nonprogressive), total motile sperm count (TMSC) and sperm morphology were analyzed. In order to determine whether the improvement in semen parameters after laparoscopic varicocele ligation occurred due to natural variability or as a result of surgery, statistical evaluation of the means was performed using the Wilcoxon test in case of non-normal distribution and t‑test for paired samples in case of normal distribution. Testing for normal distribution was performed by using Shapiro-Wilk test. Due to the small number of cases in group 2, the evaluation was carried out using descriptive statistics.

## Results

We identified 27 patients who were included in the analysis. The mean age was 30 years (range 18–41 years, standard deviation ± 6 years). All patients had grade II (37%) or grade III (59%) varicocele. Most patients presented with an isolated left-sided varicocele (67%). Nearly one third of patients were diagnosed with bilateral varicocele (30%). Isolated right-sided varicocele did not occur in the population. Preoperative endocrinological diagnostics showed normogonadism or hypogonadism. Patients with hypogonadism showed normal or elevated gonadotropins in the sense of normogonadotropic or hypergonadotropic hypogonadism (see Table 3). The average duration of surgery was 52 min, with the fastest operation taking 17 min and the longest taking 120 min. There were no major complications (accidental organ injury, bleeding, infection) during any of the surgeries.

**Table 3:** Patient characteristics

Age (years)	30.1 ± 6^a^
*Varicocele degree (n, %)*	
Subclinical/I°	0
II°	10 (37%)
III°	16 (59%)
*Varicocele location (n, %)*	
Left	18 (67%)
Right	0
Both	8 (30%)
Testosterone (µg/l)	4.48 ± 1.84^a^
FSH (IU/l)	8.22 ± 4.74 ^a^
LH (IU/l)	5.2 ± 2.58 ^a^

*FSH* Follicle-stimulating hormone, *LH* Luteinizing hormone

^a^ mean ± standard deviation

Of the 27 patients included in the analysis, 22 patients (81%) could be assigned to group 1 and 5 patients (19%) to group 2. The allocation was based on the preoperative spermiogram findings (see Table 4).

**Table 4:** Comparison of the preoperative and postoperative semen analyses

	Group 1 *n* (%)	Group 2 *n* (%)
*Preoperative*	22 (81)	5 (19)
*12 months postoperative*		
Azoospermia	0 (0)	4 (80)
OAT^a^	15 (68)	1 (20)
Normospermia	7 (32)	0 (0)

^a^OAT oligozoospermia and/or asthenozoospermia and/or teratozoospermia

In group 1, spermiogram diagnosis improved 12 months postoperatively for 7 patients (32%), so that these patients fulfilled the criteria of normospermia after the surgery. The initial spermiogram diagnosis persisted for 15 patients of group 1 (66%). The mean of seminal parameters in group 1 were compared before and 1 year after surgery (see Table 5). A significant improvement was found for sperm concentration (*p* < 0.005), TSC (*p* < 0.005), TMSC (*p* < 0.005) and sperm motility (*p* < 0.05). There was no significant improvement in sperm morphology (*p* > 0.05) after the treatment.

**Table 5:** Group 1 preoperative and 12 months postoperative semen parameters

Parameter	Preoperative	Postoperative	*p*-Value
*Total sperm count (TSC, 10*^*6*^*)*	43 ± 68	123 ± 110	< 0.005^a^
*Sperm concentration (10*^*6*^*/ml)*	10 ± 28	50 ± 48	< 0.005^a^
*Sperm motility (%)*	30 ± 1	39 ± 22	< 0.05^b^
*Total motile sperm count (TMSC, 10*^*6*^*)*	6 ± 16	27 ± 46	< 0.005^a^
*Sperm morphology (%)*	3 ± 20	3.5 ± 21	> 0.05^a^

All values as median ± standard deviation

^a^Wilcoxon-Test

^b^t-Test

In group 2, one patient was found to have spermatozoa in the ejaculate 12 months postoperatively. This patient showed OAT syndrome in the ejaculate analysis after surgery. The other 4 patients of group 2 showed persisting nonobstructive azoospermia in control semen analysis 1 year after surgery.

No complications according to Clavien-Dindo classification were found after surgery. At the time of control spermiogram none of the patients showed postoperative hydrocele formation or recurrence of varicocele.

## Discussion

Open surgical, microsurgical and laparoscopic techniques and percutaneous varicocele embolization are treatment options for varicoceles. First experiences with robot-assisted procedures have already been reported [13]; however, currently the most frequently used methods for varicocele treatment are microsurgical and laparoscopic varicocelectomy [8]. The postoperative improvement of seminal parameters after varicocele ligation has been reported. Numerous studies have demonstrated the positive effect on sperm concentration, motility and morphology after surgery [9, 14]. The spontaneous pregnancy rates of couples where the man has a varicocele and sperm alterations are also significantly increased after varicocelectomy [15]; however, the studies investigate different techniques and heterogeneous patient populations, so that further studies are needed for an evidence-based recommendation for laparoscopic varicocelectomy in male infertility.

Our study with new findings supports the evidence that patients with impaired seminal parameters achieve an improvement in semen quality after laparoscopic varicocelectomy. Sperm concentration, TSC, TMSC and sperm motility improved significantly after the treatment. The change in the seminal parameters is in accordance with further studies on laparoscopic varicocele ligation. In a prospective, randomized study of 94 infertile patients Al-Said et al. were able to show a comparable increase in the seminal parameters after laparoscopic varicocelectomy [9]. Unfortunately, this study did not analyze the effects of surgery on TMSC.

In comparison with microsurgical varicocelectomy, our study shows a comparable improvement of seminal parameters after laparoscopic ligation. Watanabe et al. showed an analogous increase in sperm concentration and sperm motility. Nevertheless, the authors reasoned that the microsurgical procedure was favored because of the lower rates of postoperative hydroceles and lower recurrence rates [14]. In contrast, no postoperative hydrocele and no recurrence were observed in our study and laparoscopic varicocelectomy proved to be safe and with few complications. Some authors further state that postoperative hydrocele formation after laparoscopic varicocelectomy can be reduced by lymphatic vessel-sparing techniques [16].

A postoperative normospermia showed up in 30% of the cases with at least 1 impaired ejaculate parameter preoperatively. For these patients there is the possibility to achieve pregnancy without ART after the surgery. Despite this positive aspect, OAT persisted in most of the patients. Prospective, randomized studies on varicocelectomy also showed an improvement in sperm concentration, motility and morphology [17]; however, a normalization of seminal parameters in terms of normospermia has not been reported. It can therefore be concluded that most patients with impaired semen parameters preoperatively continue to rely on assisted reproduction techniques postoperatively.

For patients with nonobstructive azoospermia, the aim of varicocelectomy is the induction of spermatogenesis and thus the postoperative detection of sperm in the ejaculate. This is to avoid sperm retrieval by testis tissue extraction (TESE) and the associated injury to the testicular parenchyma. This effect has already been demonstrated for percutaneous varicocele embolization [18]. Even if no spermatozoa are detected in the ejaculate postoperatively, the probability of successful TESE may be increased. In a systematic review of varicocele ligation in NOA, Esteves et al. reported that pregnancy occurs in 13.6% of cases after varicocelectomy. The sperm extraction rates were also significantly increased after surgery [19]. In our study, spermatozoa were detected in the ejaculate of one patient postoperatively. Although our study does not allow a quantitative statement on the induction of spermatogenesis due to the small sample size, this case shows impressively that patients with NOA can avoid a testicular surgery for sperm extraction by a laparoscopic varicocele ligation.

Various methods of artificial reproductive techniques are available to infertile couples. The spectrum of treatment ranges from invasive procedures such as in vitro fertilization (IVF) or intracytoplasmic sperm injection (ICSI) to less invasive procedures as intrauterine insemination (IUI). These methods may be preceded by TESE if the ejaculate quality is insufficient. Following laparoscopic varicocelectomy, most of our patients with both preoperative NOA and OAT remained reliant on ART. Whether these patients can use less invasive methods of ART after surgery depends in clinical practice on the improvement of TMSC [7]. In our study a significant increase of TMSC was observed for patients with impaired seminal parameters preoperatively. Thus, these patients can seek less invasive ART such as IUI after laparoscopic varicocelectomy.

Limitations of our study are the retrospective, nonrandomized and uncontrolled study design and the small sample size. Furthermore, our work does not offer a direct follow-up of pregnancy rates. Rather, the improvement of semen parameters is an indirect indicator for the probability of a pregnancy or the success of assisted reproductive measures. Therefore, further prospective, randomized and controlled trials on laparoscopic varicocelectomy in male infertility are needed for a stronger evidence base.

In conclusion, laparoscopic varicocele ligation is a valid therapeutic option to improve seminal parameters for further assisted reproductive techniques. In general, induction of spermatogenesis and postoperative detection of sperm in the ejaculate of patients with NOA can be achieved. These patients benefit from the surgery, as invasive procedures for sperm extraction can be avoided. Patients with OAT have a significantly improvement of sperm density, sperm count and sperm motility. In addition, a normospermia can be achieved. These patients may seek natural conception after laparoscopic varicocelectomy, although most patients remain dependent on assisted reproductive techniques.
